# Improved Malaria Therapy with Cationic Nanocapsules Demonstrated in *Plasmodium berghei*-Infected Rodents Using Whole Blood Surrogate Population PK/PD Modeling

**DOI:** 10.3390/pharmaceutics16111369

**Published:** 2024-10-25

**Authors:** Tamara Ramos Maciel, Ana Claudia Funguetto-Ribeiro, Laura Ben Olivo, Flávia Elizabete Guerra Teixeira, Camila de Oliveira Pacheco, Bibiana Verlindo de Araujo, Sandra Elisa Haas

**Affiliations:** 1 Pharmacology and Pharmacometric Laboratory, LABFAR, Federal University of Pampa (UNIPAMPA), Uruguaiana 97501-970, RS, Brazil; tamararmaciel@gmail.com (T.R.M.); acfunguetto@gmail.com (A.C.F.-R.); flaviateixeira02@outlook.com (F.E.G.T.); coliveirapacheco@gmail.com (C.d.O.P.); 2 Pharmaceutical Sciences Post Graduate Program, Federal University of Pampa (UNIPAMPA), Uruguaiana 97501-970, RS, Brazil; 3 Pharmaceutical Sciences Post Graduate Program, College of Pharmacy, Federal University of Rio Grande do Sul, Porto Alegre 91060-100, RS, Brazil; laura.olivo@ufrgs.br (L.B.O.); bibiana.araujo@ufrgs.br (B.V.d.A.)

**Keywords:** interspecies translation, whole blood, quinine, nanocapsules, malaria, PK/PD modeling

## Abstract

**Objectives**: Investigating how nanoparticle systems interact in whole blood (WB) is critical to evaluating the effectiveness of malaria therapy. Methods: We decided to establish a pharmacokinetic/pharmacodynamic (PK/PD) model of the quinine population in WB using *Plasmodium berghei*-infected mice, with a subsequent model comparison for nanocapsules coated with polysorbate (NCP80) or prepared with Eudragit^®^ RS (NCEUD). The WB quinine population pharmacokinetic model in rats was developed using plasma and partition coefficients for rat erythrocytes. Mouse WB quinine population PK/PD modeling was developed using allometrically scaled literature-free mouse quinine pharmacokinetic data and covariate values to obtain a WB population pharmacokinetic model for quinine and nanocapsules in mice. This allowed for PK/PD modeling of the quinine population with the WB concentration and parasitemia data in mice. All models were built in NONMEN. **Results**: The WB quinine concentration profiles in rats were characterized using a two-compartment model. Nanoencapsulation reduced clearance and central compartment volume and increased peripherical compartimental volume. A maximum effect model described the PK/PD of the quinine WB population in mice, demonstrating that NCEUD enhances the antimalarial effect. **Conclusions**: Quinine WB is a good surrogate for describing the response to exposure in malaria. NCEUD outperformed NCP80 and free quinine, suggesting that cationic surfaces improve the potential for treating malaria.

## 1. Introduction

The parasite *Plasmodium* sp. is responsible for causing malaria, which the global count for 2021 saw a total of 247 million cases and 619 thousand deaths worldwide [1]. Quinine (QN) is an antimalarial drug that acts on the erythrocytic phase; its key proposed mechanism of action is to block the formation of the hemozoin in the parasite’s digestive vacuole, causing edema, pigment agglomeration, and death of the parasite [2]. Hypoglycemia, cinchonism, and cardiotoxicity are the primary adverse effects [3,4] that hinder patient adherence to treatment [5]. Likewise, QN resistance has been reported [6], highlighting the demand for new strategies to battle against malaria, such as incorporating QN into nanoparticulate systems. The primary way resistant antimalarials is through expelling the drugs at a high rate of drug efflux by resistant parasites [7].

The most important property of a nanocarrier to be used for malaria treatment is its ability to remain in the bloodstream for a long time, thereby improving its interaction with erythrocytes and the parasite’s membrane [4]. Also, targeting the drug at *Plasmodium* sp. during the erythrocytic phase has the potential to address drug resistance and serve as an alternative treatment for severe malaria [8,9]. Nanotechnology provides effective solutions for combatting the resistance of malaria parasites to conventional drugs [10,11]. By improving the drug targeting [12,13], prolonging their therapeutic action [4,11,13], and combining effective therapies [13,14], nanocarriers are becoming a promising approach for treating resistant and severe malaria infections [10,11,15].

Passive drug delivery mechanisms aimed at nanocarriers take advantage of specific tissue characteristics to target drugs [16]. Then, electrostatic interactions between the surface charges of erythrocytes and nanocarriers can be one strategy for increasing the efficacy of nanoparticles in malaria [17]. In this case, the surface of nanocapsules (NC) can be modified to improve the biological performance of drugs [18,19,20,21].

Our research team has dedicated efforts to enhancing the efficacy of QN by nanoencapsulating it, resulting in promising findings. QN-Loaded NC-coated polysorbate 80 (NCP80), an anionic surface system, resulted in a 100% survival rate in *P. berghei*-infected rats, representing an almost 30% reduction in the effective dose (105 and 75 mg/kg/day for free and NCP80, respectively) [20]. Both NCP80- and QN-loaded NC prepared with Eudragit RS100^®^ as a polymer (NCEUD), a cationic nanoparticle, increased the QN penetration into erythrocytes infected with *Plasmodium berghei* in Wistar rats [20,22]. Both anionic (NCP80) and cationic (NCEUD) formulations showed more significant antimalarial effects than free QN, suppressing parasitemia and increasing the survival time in Peter’s four-day suppressive test against *P. berghei*-infected mice [17,19,23]. Non-compartmental plasma pharmacokinetics (PK) in *P. berghei*-infected rats showed that nanoencapsulation prolonged t_1/2_ and increased volume of distribution at steady state (Vd_ss_) for anionic NC (NCP80). For cationic NCEUD, an increase in t_1/2_ and a reduction in Vd_ss_ were observed. The Population PK modeling showed that NCP80 was a covariate for intercompartmental clearance (Q), reducing it, and for peripherical compartmental volume (V_2_), increasing it, while NCEUD influenced decreasing Q and reducing central compartmental volume (V_1_) [19].

Nevertheless, the previous studies that utilized the QN plasma concentration failed to clarify the impact of NC surface charge on QN whole blood and its effectiveness. Furthermore, they employed diverse species. In light of this, the study aimed to devise a population pharmacokinetic/pharmacodynamic (PK/PD) model for QN in Whole Blood (WB) in *P. berghei*-infected mice, comparing properties QN-loaded and NC-coated with distinct surface charges.

## 2. Materials and Methods

### 2.1. Data Used for Analysis and General Procedures

The original data used in this study were collected from our previous investigation [19]. In summary, NC suspensions were prepared by the nanoprecipitation method, which consisted of an organic phase composed of QN, caprylic/capric triglyceride, Lipoid^®^ S45 (Lipoid AG, Ludwigshafen, Germany), polymer, acetone, and an aqueous phase containing polysorbate 80 (P80) (Tween^®^ 80, Croda International PlC, Snaith, UK) and distilled water. The difference between NCEUD and NCP80 was the type of polymer used in the organic phase. Eudragit RS100^®^ (Evonik Industries AG, Essen, Germany) and poly(ε-caprolactone) (Sigma Aldrich Ltd, São Paulo, Brazil) (average Mw~14,000, average Mn ~10,000 by GPC) were added to prepare NCEUD and NCP80, respectively. Once the phases were solubilized, they were mixed. The organic solvent and water were evaporated using a rotary evaporator until the final QN concentration was in the 2 mg/mL formulation. The NC was characterized regarding the particle size, zeta potential, pH, dose content, and encapsulation efficiency.

The QN partition coefficient to erythrocytes (Kp) was evaluated using blood from *P. berghei*-infected rats with 9.25 ± 1.5% parasitemia [19]. After the erythrocyte separation and washing procedure, the hematocrit was adjusted to 0.48 by resuspending the erythrocytes in a 5% glucose solution (pH 7.4). Free QN solution, NCP80, or NCEUD were added to the erythrocyte suspension, and after incubation and centrifugation, QN quantification was performed. The QN concentration was determined using both supernatant and sediment. The Supplementary Material presents the equation calculating Kp (Equation (S1)).

The PK evaluation was performed in male Wistar rats infected with *P. berghei* on day 7 after infection (*n* = 6 per group). The animals received a single dose of 20 mg/kg of QN (iv bolus). Blood samples were collected at 0.083, 0.25, 0.5, 1, 2, 4, 6, 8, 12, 18, and 24 h after administration, and QN plasma concentrations were quantified by HPLC-MS [19], with a lower limit of quantification (LLOQ) of 10 ng/mL. No concentration below the lower limit of quantification was used.

The antimalarial efficacy (PD data) was assessed by Peters’ suppressive four-day test against *P. berghei*-infected mice. Animals were infected on day 0 (D0) and received 20 mg/kg twice a day (intraperitoneal route (ip)) from D0.5 to D3.5. The evaluated groups were NCP80, NCEUD, free QN, and saline (*n* = 5 per group). Parasitemia and the survival time of the animals, the percentage of mean parasitemia, and the antimalarial activity were evaluated. The percentage of parasitemia was evaluated microscopically after inoculation every other day until the death of all animals or D15. The parasitemia-suppressing activity was determined on the fifth day after infection by subtracting the percentage of parasitemia in the control and treated groups by the percentage of parasitemia in the treated group [19] (Supplementary Material, Equation (S2)).

### 2.2. Quinine Whole Blood Population PK Modeling in Rats

The plasma concentrations of free and nanoencapsulated QN were previously evaluated by our research group [19]. Whole Blood concentrations were estimated using the values of the QN Kp multiplied by the plasma concentration each time, resulting in the blood concentration versus time profile. The partition coefficients used were 3.74 for free QN, 8.92 for NCP80, and 6.48 for NCEUD [20]. The Area Under the Curve (AUC) was calculated using the linear Trapezoidal rule on Microsoft Excel from the whole blood concentration versus time profiles. Statistical analysis was performed to compare plasma with whole blood (one-way ANOVA, *p* < 0.05).

The QN concentration data in the whole blood versus time was included in the database. No concentration was below the lower limit of quantification. The population PK was performed using a Nonlinear Mixed-effects Modeling approach with the NONMEN software (version 7.4, ICON Development Solutions, Ellicott City, MD, USA) and PsN version 5.3.0 software (Perl-speaks-NONMEM, Uppsala, Sweden). Model parameters were estimated using the first-order conditional estimation and interaction (FOCE-I) method. The Ggplot package and lattice libraries for R, version 4.4.1, and RStudio, version 30.4.0 (The R Foundation for Statistical Computing, Vienna, Austria), were used to process the data. The error models additive, proportional, and mixed types were used to characterize the residual variabilities.

The evaluated covariates were identified through univariate research and were selected if their addition decreased by 3.84 in the objective function value (OFV) (*p* < 0.05). Significant covariates were kept in the model if their elimination could increase by at least 6.63 in OFV (*p* < 0.01). In addition, covariates with a potential physiological correlation with the parameters were maintained [21,22,24]. Formulations were evaluated as categorical covariates (free QN, NCP80, and NCEUD) or NC (NCP80 and NCEUD). The best model was selected using OFV, a visual predictive check (VPC), goodness-of-fit (GOF) plots, shrinkage analysis, and %RSE.

### 2.3. Pharmacokinetic Translation from Rats to Mice

To build the PKPD model, since the PK studies were performed in rats and the pharmacodynamic (PD) studies were carried out in mice, it was necessary to use allometric scaling to translate the exposures observed in each species, respectively, and apply this information to associate the observed effect with the concentrations predicted by allometry.

Thus, the expected concentrations in the infected mice that received free quinine, NCEUD, or NCP80 were calculated through allometry using Equation (1) [25].

(1) $$P=a\times WT^{b}$$

where *P* is the parameter of interest, *WT* is the body weight, and *a* and *b* are the coefficient and exponent of the allometric equation, respectively.

The concentrations observed in rats (from our study) and mice (from the literature [26]) were modeled simultaneously, and the allometric coefficient and exponent were determined for the pharmacokinetic parameters clearance (CL), V_1_, V_2_, and Q. In this procedure, a bioavailability in mice of 100% was assumed, which was corroborated by the intravenous (iv) and ip administration studies of two previous studies published in the literature in healthy mice [26,27].

The average profile was simulated to estimate a mice population (*n* = 10) with different weights (±1 g) and then obtain the allometric value for the population for each pharmacokinetic parameter.

### 2.4. Population PK/PD Modeling in Mice

Michels et al. [19] obtained antimalarial efficacy data using a parasitemia suppression model in mice [27]. As we performed allometry from rats to mice for the PK of QN, a simulation of the PK profile of mice was performed according to the treatment protocol, from Day 0.5 to 3.5, 20 mg/kg. These data were then modeled using NONMEN (version 7.4, ICON Development Solutions, Ellicott City, MD, USA).

Thereafter, for population PK/PD, model selection was also conducted similarly for population PK. Through OFV assessment, the precision of parameter estimates, the physiological significance of the results, and the visual assessment of standard goodness-of-fit plots were performed. Interindividual variability is considered a multiplicative effect, and normal distribution and residual variability are included in the constant error model. Covariates were evaluated similarly as those described in Section 2.2. The reliability of individual parameter estimates and goodness-of-fit plots were assessed for η and ε shrinkages.

## 3. Results

### 3.1. Plasma to Whole Blood Pharmacokinetics in Rats

**Figure 1:**
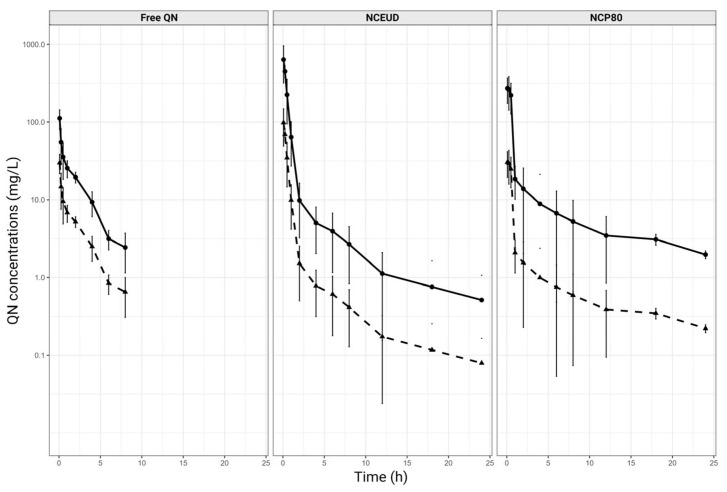
QN concentration versus time in plasma (dashed line) and whole blood (continuous line) (mean ± standard deviation) in *Plasmodium berghei*-infected male Wistar rats (20 mg/kg, iv bolus) for free QN, NCP80, and NCEUD (*n* = 6 per group).

All rats from our previously reported study (*n* = 18) were included in the analysis [19]. To obtain total QN concentrations in whole blood, plasma concentrations of free or nanoencapsulated QN (NCP80 and NCEUD) were multiplied by the Kp. Figure 1 shows that whole blood’s QN concentrations are higher than those of plasma for all groups.

The AUC_0–∞_ in whole blood was 127.8 ± 16.5, 330.9 ± 139.1, and 397.9 ± 123.9 µg·h/mL (mean ± standard deviation) for free QN, NCP80, and NCEUD, respectively. Numerically, there was an increase in AUC_0–∞_ for all treatments compared to plasma (Figure 1). The plasma AUC_0–∞_ data were 33.8 ± 4.8, 61.1 ± 41.2, and 59.8 ± 22.2 µg·h/mL for free QN, NCP80, and NCEUD, respectively [19]. Then, the plasma-to-whole blood ratio was QN > NCP80 > NCEUD (0.26, 0.18, and 0.15), showing that nanoencapsulation, regardless of NC surface charge, exhibited better exposure than free QN in whole blood (*p* < 0.05) (Figure 1).

### 3.2. Whole Blood Population PK Modeling in Rats

**Figure 2:**
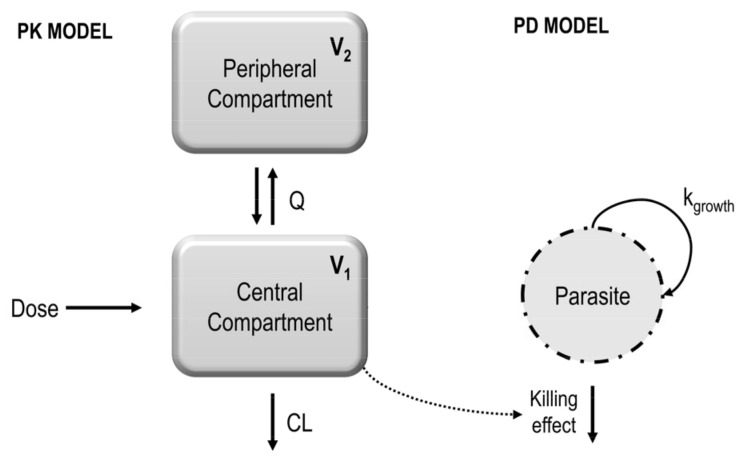
Quinine population PK/PD model structure in *P. berghei*-infected mice. V_1_ is the volume of distribution in the peripheral compartment, V_2_ is the volume of distribution in the peripheral compartment, Q is the intercompartmental clearance between the central and peripheral compartments, CL is the clearance, k_growth_ is the parasitic growth rate, and the killing effect is the parasite death through the drug concentration in whole blood.

The initial data on QN plasma levels served as the foundation for constructing a population PK model using whole blood to enhance predictions related to QN’s impact on malaria through erythrocytic activity (Figure 2). Then, 197 observations of whole blood concentrations versus the time from free and nanoencapsulated QN groups were used.

**Figure 3:**
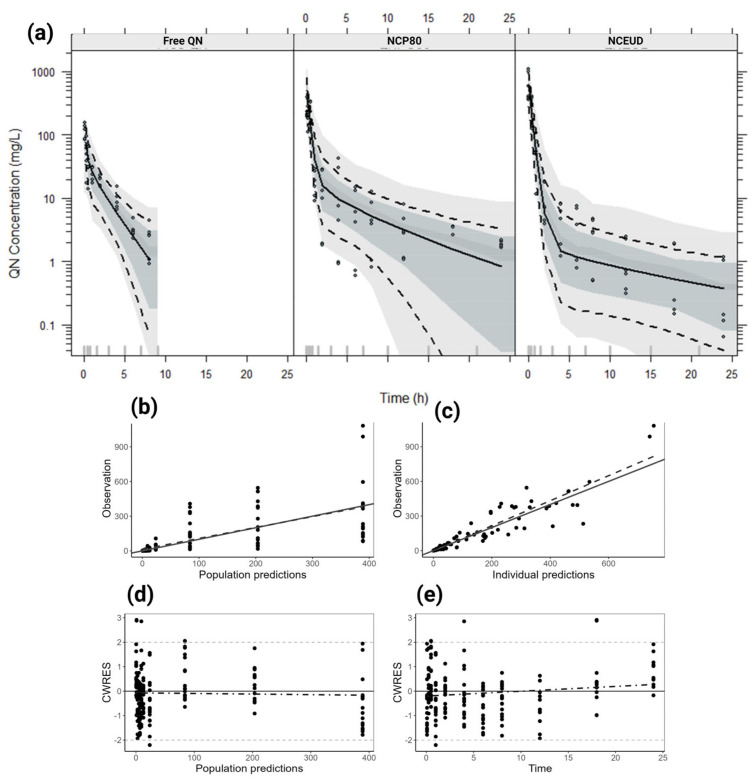
Goodness-of-fit plots of the final population PK model. (**a**) Visual predictive check (VPC) stratified by the formulation of the final population PK based on 1000 simulations. VPC shows a comparison of the observations (dots) with the 10th, 50th, and 90th percentiles of simulated profiles (shadow areas) and of observations (dashed and solid lines). (**b**) Correlation between observations and population predictions by the final model. (**c**) Correlation between observations and individual predictions by the final model. (**d**) CWERS (conditional weighted residuals) vs. individual prediction distributed around slope zero. (**e**) CWERS vs. time, showing no major bias in the model.

After comparing the structural models, evaluating the lower value of OFV, and visualizing the goodness-of-fit graphs (Figure 3), the final population PK model was a two-compartment model with first-order elimination (Equations (2)–(4)), and observation models were proportional residual error with normal distribution.

(2) $$\frac{dXc_{1}}{dt}=\left(\frac{Q}{V_{2}}\times Xc_{2}\right)-\left(\frac{Q}{V_{1}}\times Xc_{1}\right)-\left(\frac{CL}{V_{1}}\times Xc_{1}\right)$$

(3) $$\frac{dXc_{2}}{dt}=\left(\frac{Q}{V_{1}}\times Xc_{1}\right)-\left(\frac{Q}{V_{2}}\times Xc_{2}\right)$$

(4) $$\frac{dE}{dt}=\frac{CL}{V_{1}}\times Xc_{1}$$

where Xc_1_ is the amount of the drug in the central compartment, Xc_2_ is the amount of the drug in the peripheral compartment, V_1_ is the volume of distribution in the central compartment, V_2_ is the volume of distribution in the peripheral compartment, Q is the intercompartmental clearance between the central and peripheral compartments, CL is the clearance, E is the drug quantity eliminated, and t is time.

Table 1 shows the PK parameters and their corresponding interindividual variability, demonstrating that all parameters exhibited adequate relative standard errors. The initial model’s OFV, without the formulation as a covariate, was 965.75. When the NC covariate was added to the CL and V_1_ parameters, the OFV value was reduced to 953.81 and 940.7, respectively. Adding NCP80 and NCEUD in V_2_ reduced the OFV to 930.71. This reduction indicates, together with the other parameters, that the formulation is a covariate, explaining the interindividual variability of the PK parameters.

The values of CL and V_1_ were influenced by nanoencapsulation, but the types of coating do not differ from each other. Therefore, nanoencapsulation was a covariate for these parameters, demonstrating a reduction in CL to 0.059 mL/h (0.156 mL/h for free QN) and in V_1_ to 0.028 mL (0.261 ml for free QN). The surface charge impacted the V_2_ parameter, 0.204 mL for NCP80 and 0.099 mL for NCEUD for free QN. The Shrinkage-η values obtained by conditional distribution plots showed 20.2, 60.6, 12.8, and 6.9% on interindividual variability for CL, V_1_, V_2_, and Q. The ε-shrinkage was 8.3%. The VPC is shown in Figure 3.

### 3.3. Rats-to-Mice Translation Whole Blood Pharmacokinetics

**Figure 4:**
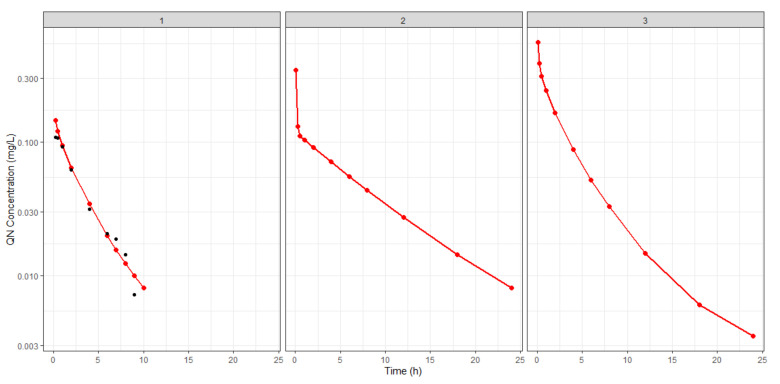
Allometric scaling for mice, proving that allometric scaling can predict Pussard et al.’s (2003) data [26] (black dots) for free QN (1) in mice. Simulated profiles regarding the covariates’ effect on the PK profile for NCP80 (2) and NCEUD (3) are shown.

To perform population PK/PD modeling and considering that PK and PD were performed in different species, it was necessary to translate the PK parameters from rats to mice allometrically. Allometric scaling was performed using the mean QN profile in whole blood in mice infected with *P. berghei* extracted from Pussard and colleagues [26]. From this, it was possible to perform the prediction of the PK profile in mice for NCP80 and NCEUD, since the covariates obtained in PK in rats were additive; the allometric scaling from rats to mice is shown in Table 2 and Figure 4.

### 3.4. Population PK/PD Analysis

**Figure 5:**
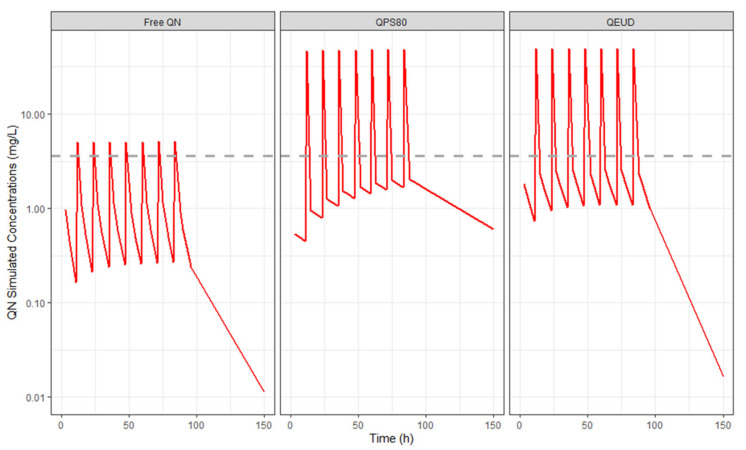
Simulation of the mouse dosage regimen to evaluate antimalarial activity. The animals were infected with D0 and received 20 mg/kg twice daily (ip) from D0.5 to D3.5. The groups evaluated were NCP80, NCEUD, and free QN. The dotted line indicates EC_50_.

Since rat-to-mouse allometry of the QN PK was necessary, a simulation of the mice’s PK profile was performed according to the treatment protocol, as shown in Figure 5.

**Figure 6:**
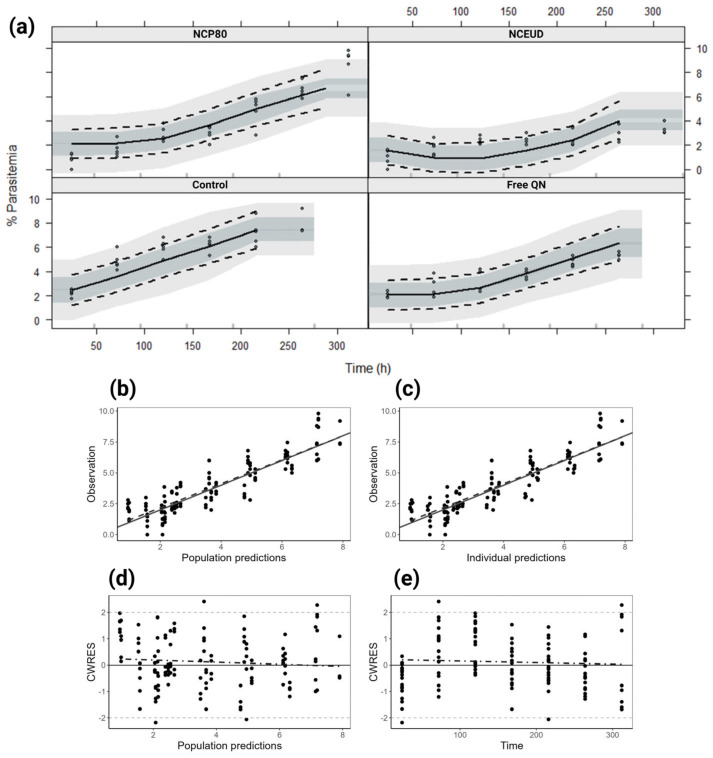
Goodness-of-fit plots of the final population PK/PD model. (**a**) Visual predictive check (VPC) stratified by the formulation of the final population PK/PD based on 1000 simulations. VPC shows a comparison of the observations (dots) with the 10th, 50th, and 90th percentiles of simulated profiles (shadow areas) and of observations (dashed and solid lines). (**b**) Correlation between observations and population predictions by the final model. (**c**) Correlation between observations and individual predictions by the final model. (**d**) CWERS (conditional weighted residuals) vs. individual prediction distributed around slope zero. (**e**) CWERS vs. time, showing no major bias in the model.

The maximum effect model (E_max_) (Equation (5)) populational PK/PD adequately described 147 observations of parasitemia of free QN, NCP80, and NCEUD in mice (Figure 6). The parameters were estimated, and the categorical covariate formulation was significant in the E_max_ (Table 3). The VPC graph demonstrates the model’s stability (Figure 6a). The model was validated through the fit adequacy graphs (Figure 6b–e) and OFV (128.79) assessment (Table 3).

(5) $$\frac{dN}{dt}=\left\{\left[k_{g}\left(1-\frac{N}{N_{max}}\right)\right]-\left.\frac{E_{max}\times C}{{EC}_{50}+C}\right\}\right.\times N$$

where N is the number of parasites at time zero or inoculum (10^8^ *P. berghei*-infected erythrocytes), k_g_ is the parasitic growth rate, N_max_ is the maximum number of parasites able to infect the animals before their death, E_max_ is the maximum effect, EC_50_ is the concentration required for 50% of the maximum effect, and C is the drug concentration in the whole blood.

The best model for describing the effect of QN considers parasitic growth in the absence of the drug (N) as a function of time, which determines the parasitic growth constant (k_g_) based on the initial inoculum (N0). When starting treatment, the drug negatively influences the parasitic generation constant, and the antiparasitic effect can be determined (E_max_). The results are listed in Table 3. Parasitemia data as a function of time for animals treated with saline (negative control) were added to the datasheet to obtain the growth curve in vivo. The ε-shrinkage was 0.3%. The OFV of the model without covariates was 155.68; adding the NCP80 and NCEUD formulations as covariates in the E_max_ parameter reduced the value to 128.79.

The test of formulation as a categorical covariate was investigated to see if it could improve the data fit and explain the inter-individual variability. The formulations influenced the E_max_ parameter, increasing the population E_max_ from 0.055 to 0.025 and 0.069 for NCP80 and NCEUD, respectively. This demonstrated a superior effect for NCEUD compared to the free quinine.

## 4. Discussion

Reducing the volume of distribution of the central compartment seems to be fundamental for antimalarial activity since, with a smaller volume of distribution, the drug remains longer in the central compartment where the parasite is present, thus demonstrating better efficacy [28,29,30]. The accuracy of drug concentration assessments using plasma may be affected by the lack of consideration for partitioning with erythrocytes [28], which is pivotal for antimalarial drugs. While free drugs are well understood, clarity is absent in understanding the behavior of nanoencapsulated drugs.

Population PK and population PK/PD models describe the relationship between the pharmacological characteristics and observed exposure [31], and they are playing a key role in improving malaria treatment [32]. Furthermore, these models are useful in developing, optimizing, and understanding the possibilities and limitations of nanotechnology for drug delivery, allowing this approach to be applied to improve clinical and market rates [33]. Due to these factors, we have chosen to employ these tools for cross-species comparison using rodent data.

As expected, the AUC_0–∞_ of QN in whole blood showed higher values for all formulations compared to the plasma [19]. Pussard et al. [26] reported that the AUC_0–∞_ in whole blood of free QN was 36.6 ± 13.6 µg·h/mL for mice after 80 mg/kg (ip). Our approach assumes that the erythrocyte partition coefficients in *P. berghei*-infected rodents are suitable for translating PK from plasma to whole blood, independent of rats or mice. The method for determining the QN partition coefficient for erythrocytes is in vitro and has limitations, mainly related to the technique. However, it is an important alternative since the quantification of many whole blood samples also has major limitations, such as the complexity of this matrix compared to plasma [34].

The best structural model for QN in plasma [19] and whole blood in rats was a two-compartment model with first-order elimination. In plasma, the NCEUD formulation reduced V_1_ and Q, while the NCP80 reduced Q and increased V_2_ [19]. When evaluating the data separately for whole blood, we observed no differences between the NC formulations for these parameters. Therefore, we added the NC covariate only to differentiate it from the free formulation, which explained the interindividual variability. For V_2_, we observed a difference not only between the NC and the free formulation but also between the NC formulations. Therefore, for this parameter, the covariates were broken down into NCP80 and NCEUD. Both formulations increased V_2_, as was observed for plasma. Malaria reduces QN tissue disposition, probably due to increased QN binding to alpha-1-acid glycoprotein [35] and an increased uptake of erythrocytes [19]. The nanoencapsulation was able to increase tissue distribution.

The positive charge of NCEUD increased the QN interaction and its specificity with erythrocytes due to the negative charge of blood cells [36,37]. Our study’s premise is that the infection level is the same in rats and mice; thus, the species is not a determining factor for the disease.

Our study showed that the maximum effect of QN was superior compared to the free drug. This was evidenced by comparing the graphs of the percentage of parasitemia as a function of time, in which parasitemia always occurs lower in other groups. In addition, the survival time of the animals was also superior [19]. Other studies have also shown that nanoencapsulation improves the effectiveness of antimalarial drugs. Sari et al. (2018) developed and evaluated andrographolide-containing carboxymethyl chitosan nanoparticles and observed that the nanoparticles improved antimalarial activity in *P. berghei*-infected mice due to chitosan improving the dissolution of andrographolide by altering its physical state and decreasing its melting point and degree of crystallinity [38]. Ismail and coworkers demonstrated that artemisinin-loaded liposomes increased parasite death in *P. berghei*-infected mice, delayed recurrence, and increased animal survival rates compared to the free drug; the authors attributed the improved effect to the smaller volume of distribution and thus provided more time to be internalized in infected erythrocytes [39]. The effects of coating NC on curcumin properties were evaluated by Santos et al., the researchers noted that P80, EUD, polyethylene glycol, or chitosan-coated NC demonstrated superior antimalarial activity when compared to free curcumin, with a special mention of the chitosan-coated formulations and their cationic surface charge [18].

In developing antimalarial drugs, preclinical studies in rodents are essential, despite the difficulties, especially regarding the heterogeneity between the presentation of malaria in rodents [40] or primates [41] and that in humans. This large spectrum of the disease in the different models may reflect the diversity of the disease and may be considered a strength rather than a limitation [40]. Therefore, when translating these PK and PD models of malaria from rodents to humans, it should be considered that the disease may have a different progression and different disease-causing parasites.

Nanoparticle systems are very complex, and there may be many differences between the type of systems depending on whether they involve inorganic or organic nanoparticles, which have different characteristics and can considerably impact the PK and PD of the drug carried [42]. In our study, the surface charge proved to be very important in this context, as observed in the study by Funguetto-Ribeiro et al. (2023) regarding the coating with chitosan. This polymer confers a positive surface charge for lipid-core NC containing clozapine, which led to an increase in the number of compartments in the PK of the NC with this coating compared to the others, in addition to a decrease in V_1_ and a higher V_2_ [21], the same behavior observed for our NC formulations. For Carreño et al. (2020), the key point for improving the PK of quetiapine was the distribution of the drug in the pseudophases of the nanocapsules. The authors observed that quetiapine NCs could release the drug into the plasma with two distinct first-order rate constants, depending on whether the drug was dispersed in the core or adsorbed on the polymeric wall. They demonstrated that nanoencapsulation altered the PK and the effect of quetiapine compared to the free one [43,44]. For Lebreton, there was an influence of the size and coating on the PK properties of intact lipid nanocapsules, in which PEG-coated nanocapsules with a size of 85 nm reduced clearance, compared to those with a size of 50 nm coated with PEG or sterylamine or without any coating [45]. Therefore, these approaches are very useful tools for evaluating the preclinical performance of nanoparticles. Thus, it is possible to identify the most important characteristics within the nanotechnological system.

In conclusion, our findings indicate that whole blood is a good surrogate for describing the exposition-response in malaria at the fixed dose evaluated. Cross-species translation from rats to mice allowed for obtaining a population PK/PD model. The cationic nanocapsules (NCEUD) were more effective than anionic nanocapsules (NCP80) against *P. berghei*-infected mice. In addition, this study showed the pharmacometrics’ applicability in screening nano-based formulations at the pre-clinical stage.

## Figures and Tables

**Table 1:** QN parameter estimates from the final population PK model in whole blood in *Plasmodium berghei*-infected rats.

Parameter	Estimate (RSE%)	Interindividual Variability (CV%)	Shrinkage-η (%)
CL__pop_ (mL/h)	0.156 (9.0)	8.5 (32.4)	20.2
CL_NC (mL/h)	0.059 (15.5)	-	-
V_1___pop_ (mL)	0.261 (10.2)	1.3 (120.1)	60.6
V_1__NC (mL)	0.028 (11.5)	-	-
Q__pop_ (mL/h)	0.025 (25.3)	89.8 (30.0)	6.9
V_2___pop_ (mL)	0.066 (49.9)	184.4 (29.3)	12.8
V_2__NCP80 (mL)	0.204 (17.2)	-	-
V_2__NCEUD (mL)	0.099 (39.7)	-	-
**Residual Variability**			
**Parameter**	**Estimate (RSE%)**		
Proporcional Model	43.6 (7.3)		
OFV	930.71		

CL—Clearance; V_1_—Volume of distribution of the central compartment; V_2_—Volume of distribution of the peripheral compartment; Q—Intercompartmental Clearance; RSE%—Relative standard error (standard error of estimate/estimate × 100). Formulations were evaluated as categorical covariates using NC for two formulations (as the CL parameter) or NCP80 and NCEUD (V_2_).

**Table 2:** Allometric scaling from rats to mice for free QN and influence of nanostructures in these species.

Allometric Scaling from Rats to Mice for Free QN	Influence of Nanostructures in Rats	Influence of Nanostructures in Mice
${CL}_{SPECIE}=0.0112\times {WT}^{-1.43}$	${CL}_{NC}={CL}_{FREE}-0.0973$	${CL}_{NC}={CL}_{FREE}-1.353$
${V_{1}}_{SPECIE}=0.00227\times {WT}^{-2.06}$	${V_{1}}_{NC}={V_{1}}_{FREE}-0.233$	${V_{1}}_{NC}={V_{1}}_{FREE}-3.695$
${V_{2}}_{SPECIE}=0.031\times {WT}^{-1.22}$	${V_{2}}_{NCP80}={V_{2}}_{FREE}+0.138$	${V_{2}}_{NCP80}={V_{2}}_{FREE}+4.494$
$Q_{SPECIE}=0.0136\times {WT}^{-1.93}$	${V_{2}}_{NCEUD}={V_{2}}_{FREE}+0.0335$	${V_{2}}_{NCEUD}={V_{2}}_{FREE}+0.274$

CL—clearance; V_1_—volume of distribution of the central compartment; V_2_—volume of distribution of the peripheral compartment; Q—intercompartmental clearance.

**Table 3:** Estimated parameters for the QN population PK/PD model in *P. berghei*-infected mice.

Parameter	Estimate (RSE%)	Interindividual Variability (CV%)
k_g___pop_ (h^−1^)	0.012 (5.8)	-
E_max___pop_	0.055 (71.3)	0.008
E_max__NCP80	0.025 (77.9)	-
E_max__NCEUD	0.069 (61.3)	-
EC_50___pop_ (mg/L)	3.59 (104.2)	5.9 (8.4)
N_max_ (%) (Fix)	9.2 (0%)	-
**Residual Variability**		
**Parameter**	**Estimate (RSE%)**	
Additive Error (mg/L)	−0.933 (10.7)	
Proporcional Error (%)	9.34 (55.2)	
OFV	128.79	

RSE%—relative standard error (standard error of estimate/estimate × 100); CV%—coefficients of variation; N—number of parasites (*P. berghei*-infected erythrocytes); k_g_—parasitic growth rate; N_max_—maximum number of parasites in the absence of the drug; E_max_—maximum effect; EC_50_—concentration required for 50% of the maximum effect; C—drug concentration in the whole blood; OFV—objective function value. Formulations were evaluated as categorical covariates (NCP80 and NCEUD).

## Data Availability

The raw data supporting the conclusions of this article will be made available by the authors upon request.

## Supplementary Materials

The following supporting information can be downloaded at: https://www.mdpi.com/article/10.3390/pharmaceutics16111369/s1, Equations (S1) and (S2). References [19,23,46] are cited in the Supplementary Materials.
