# Supplementary material for: Improved Malaria Therapy with Cationic Nanocapsules Demonstrated in Plasmodium berghei-Infected Rodents Using Whole Blood Surrogate Population PK/PD Modeling
Source: Pharmaceutics. 2024 Oct 25;16(11):1369. doi: 10.3390/pharmaceutics16111369 (PMC11597719; doi:10.3390/pharmaceutics16111369)
Supplement: Supplementary file 1 [file pharmaceutics-16-01369-s001.zip › pharmaceutics-3090866-supplementary.pdf]

Supplementary Material

Data used for analysis and general procedures

a. Determination of the partition coefficient [1-3]

The partition coefficient of QN (K_P) was determined by Equation S1.

$$K_p = \frac{A_S - (C_{sup}.V_S.(1-H))}{H.V_S.C_{sup}} \quad \text{S1}$$

Where, A_s is the drug concentration added to the medium (10 µg/mL), C_{sup} is the drug concentration in the supernatant, V_s is the final suspension volume of erythrocytes (1 mL), and H is the hematocrit (0.48).

b. Evaluation of parasitemia variation[3]

$$Variation\ of\ parasitemia = 100 \times \left(\frac{Control - Treated}{Treated}\right) \quad \text{(S2)}$$

Where *Control* is the mean percentage of parasitemia in the control group and *Treated* is the mean percentage of parasitemia in the treated group.

References

1. Derendorf, H.; Gramatté, T.; Schäfer, H.G. *Pharmakokinetik: Einführung in die Theorie und Relevanz für die Arzneimitteltherapie ; 29 Tabellen*; Wiss. Verlag-Ges.: 2002.
2. Haas, S.E.; Bettoni, C.C.; de Oliveira, L.K.; Guterres, S.S.; Dalla Costa, T. Nanoencapsulation increases quinine antimalarial efficacy against Plasmodium berghei in vivo. *Int J Antimicrob Agents* **2009**, *34*, 156-161, doi:10.1016/j.ijantimicag.2009.02.024.
3. Michels, L.R.; Maciel, T.R.; Nakama, K.A.; Teixeira, F.E.G.; de Carvalho, F.B.; Gundel, A.; de Araujo, B.V.; Haas, S.E. Effects of Surface Characteristics of Polymeric Nanocapsules on the Pharmacokinetics and Efficacy of Antimalarial Quinine. *Int J Nanomedicine* **2019**, *14*, 10165-10178, doi:10.2147/IJN.S227914.
